# Ruthenium (II) Complexes Based on Phenanthroline-Tetrazole as Possible Anticancer Agents

**DOI:** 10.5812/ijpr-136738

**Published:** 2023-09-07

**Authors:** Saeid Abaspour, Behzad Soltani, Hamed Hamishehkar, Moayad Hossaini Sadr

**Affiliations:** 1 Department of Chemistry, Faculty of Science, Azerbaijan Shahid Madani University, Tabriz, Iran; 2 Drug Applied Research Center, Tabriz University of Medical Sciences, Tabriz, Iran

**Keywords:** Ruthenium (II) Complexes, Tetrazole Ligand, Anticancer, Apoptosis, Autophagy

## Abstract

**Background:**

The development of platinum-based metal complexes in oncology is limited due to vigorous toxicity and drug resistance.

**Objectives:**

This work aimed to study the cytotoxic activity and apoptosis induction of ruthenium complexes in a B16F10 cell line therapy.

**Methods:**

We prepared a series of innovative Ru(II) complexes [Ru(Tzphen)(bpy)(dcbpy)]^+2^ (S1), [Ru(dcbpy)_2_(Tzphen)]^+2^ (S2), [Ru(Phen)_2_(Tzphen)]^+2^ (S3), [Ru(Tzphen)(bpy)_2_]^+2^ (S4), [Ru(dmbpy)_2_(Tzphen)]^+2^ (S5) based on 1,10-phenanthroline ligand containing tetrazole and their anticancer properties investigated by cytotoxicity in vitro, reactive oxygen species, apoptosis with annexin V/PI staining method, autophagy, and cell uptake.

**Results:**

S1, S2, S3, S4, and S5 complexes showed comparable cytotoxicity activity relative to cisplatin against the B16F10 model. Moreover, intracellular ROS levels increased due to the presence of the complexes. Among the investigated complexes, the cells treated with the S5 complex indicated the highest apoptotic percentage (Q3) of 14.9% compared to the controls. The cell adsorption of the complexes also showed that the S4 and S5 complexes had higher cell adsorption, better internalization, and higher fluorescence light intensity.

**Conclusions:**

The present work provides important guidance for designing and using Ru complexes in cancer therapy.

## 1. Background

In recent decades, platinum complexes have been used extensively in oncology. However, due to the need for solubility, vigorous toxicity, and drug resistance, the clinical usage of platinum-based complexes is limited (1-7). To overcome these problems, many efforts have been made to develop alternative metal-based compounds as antitumor candidates. There is much emphasis on ruthenium complexes as alternative compounds due to their predictable geometry, including alternative metals with variable oxidation states, rich photochemical and photophysical properties, and low toxicity to natural cells (8-10). The Ru(II) complexes based on polypyridyl ligands have made great strides in bioactivity due to their strong immunogenicity and various cytotoxic properties. Hence, they offer a novel approach for sketching metal-based antitumor drugs with advanced activity (11-14). Chen et al. showed that the polypyridyl ruthenium complexes, including N, N-chelating ligands, a complex with strong antiproliferative activity, are allowed to compel mitochondrial-induced and caspase-dependent apoptosis in human cancer cells (15, 16). Gill et al. showed that modifying lipophilicity and cell uptake by ligand modification significantly affected the cytotoxicity and intercellular targets of the Ru(II) complex (17).

Moreover, heterocyclic compounds have been used to treat many diseases, such as cancer (18, 19). Drugs containing heterocyclic rings in their structure, such as indoles, benzothiazole, camptothecin, and benzimidazole, are considered for use for anticancer purposes (20, 21). Tetrazole, as a heterocyclic structure, has recently been the focus of research, and several studies have proven its applications in medicine (22). Tetrazole-containing derivatives have demonstrated anti-hypertension, anti-fungal, anti-tuberculosis, antimalarial, anti-leishmaniasis, anti-diabetic, and anticancer activities (22-26). Recently, Yang et al. reported that the ruthenium complexes, including tetrazole moiety, can prevent cell proliferation in vitro and may be potential candidates for photodynamic therapy (27).

**Figure 1.**
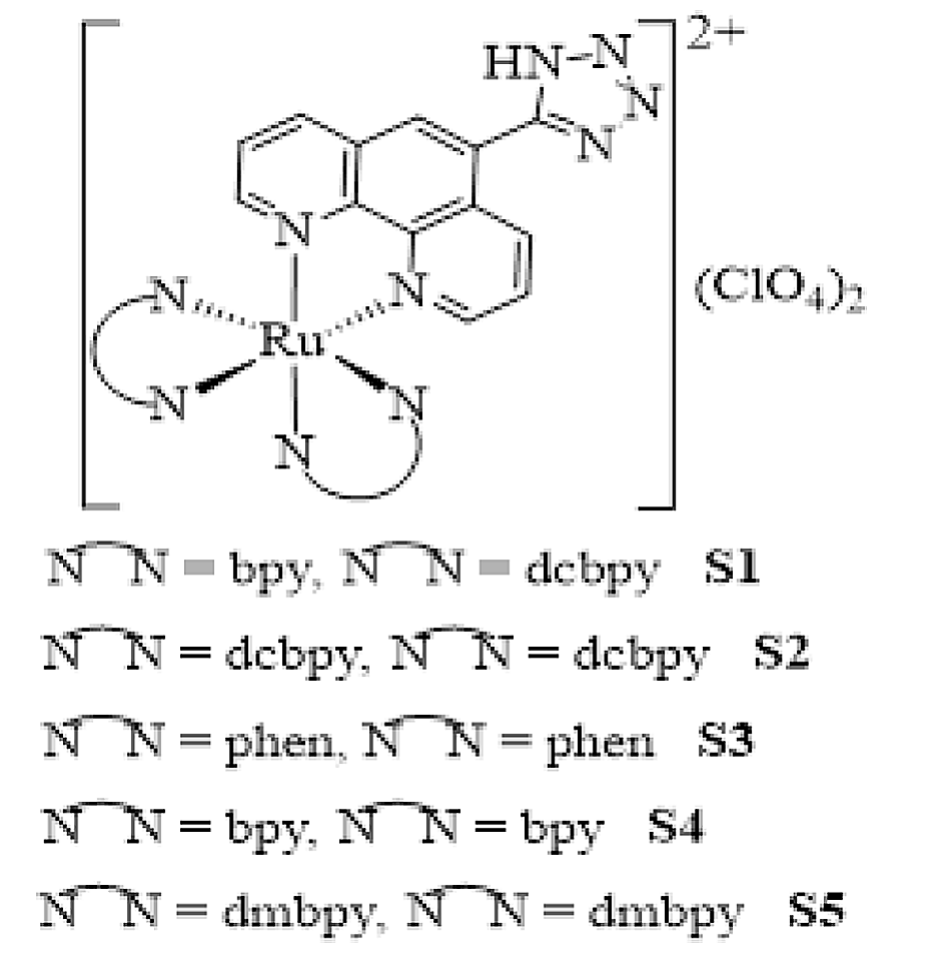
The structure of ruthenium complexes used in this study

Herein, for more insight into the anticancer activity of Ru(II) complexes, a series of innovative Ru(II) complexes based on 1,10-phenanthroline ligand containing tetrazole substitution, [Ru(Tzphen)(bpy)(dcbpy)]^+2^ (S1), [Ru(dcbpy)_2_(Tzphen)]^+2^ (S2), [Ru(phen)_2_(Tzphen)]^+2^ (S3), [Ru(Tzphen)(bpy)_2_]^+2^ (S4) and [Ru(dmbpy)_2_(Tzphen)]^+2^ (S5) were synthesized based the literature protocols (28, 29) and fully investigated using spectroscopic techniques (Figure 1). The anticancer properties of the complexes were researched by cytotoxicity in vitro, reactive oxygen species, apoptosis method with annexin V/PI staining method, autophagy, and cell uptake.

## 2. Objectives

The advancement of platinum-based metal complexes in oncology is restricted due to several limitations and challenges, such as aggressive toxicity and drug resistance. This research aimed to investigate the cytotoxic activity and apoptosis-inducing capabilities of ruthenium complexes using a murine melanoma cell line as a model system.

## 3. Methods

### 3.1. Experimental Section

#### 3.1.1. Materials

RuCl_3_.3H_2_O, 4,4’-dimethyl-2,2’-dipyridyl (dmbpy), ammonium acetate, 2,2-’bipyridine (bpy), 3,4-diaminobenzophenone were purchased from Sigma Aldrich. 1,10-phenanthroline (phen) and 1,5-diaminonaphthalene were purchased from Merck. The 2,2’-bipyridine-4,4’-dicarboxylic acid (dcbpy) was prepared according to the procedures outlined in the literature (30).

#### 3.1.2. Synthesis

##### 3.1.2.1. Ligand

**Figure 2.**
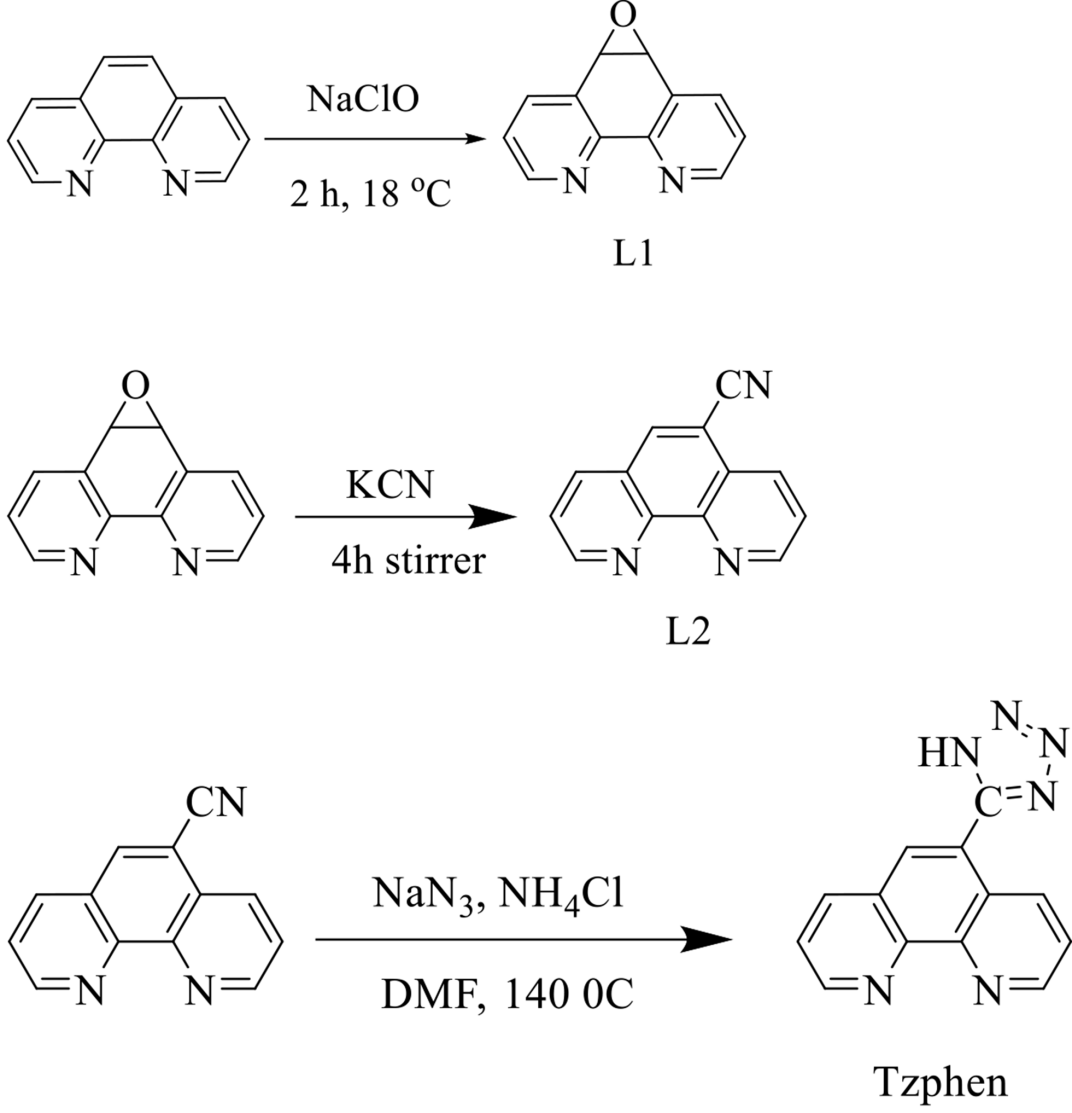
The synthesis route of Tzphen

The 5,6-epoxy-5,6-dihydro-[1,10]phenanthroline (L1), 1,10-phenanthroline-5-carbonitrile (L2), and 5-(1H-tetrazol-5-yl)-1,10-phenanthroline (Tzphen) were synthesized according to the literature protocols (28, 31-34). Briefly, L2 (410 mg, 2.0 mmol), NH_4_Cl (135 mg, 2.5 mmol), and NaN_3_ (160 mg, 2.5 mmol) in 10 mL of DMF were refluxed at 140°C for 48 h. The cooled mixture was poured into H_2_O and filtered. The filtrate was acidified to pH = 3.5 with concentrated HCl. After stirring for five hours, the suspension was filtered. The resulting solid was washed with H_2_O (2 × 5 mL) and dried in a vacuum over P_2_O_5_ at room temperature. Yield: 54%. FT-IR (cm^-1^): 3392 (m), 3069 (m), 1602 (s), 1545 (s), 1418 (w), 867 (m), 727 (w). 1H NMR (250 MHz, DMSO): *δ* 9.3 (d, J = 8.5 Hz, 1H), 9.2 (d, J = 8.5 Hz, 1H), 8.8 (s, 1H), 8.6 (d, J = 6.5 Hz ,1H), 8.65 (d, J = 6.5 Hz,1H), 7.9 (t, J = 5.5 Hz, 1H), 7.8 (t, J = 5.5 Hz, 1H). Anal. Calcd for C_13_H_8_N_6_: C, 56.722; H, 4.032; N, 30.533. Found: C, 56.731; H, 4.041; N, 30.545 (Figure 2). 

##### 3.1.2.2. Complexes

**Figure 3.**
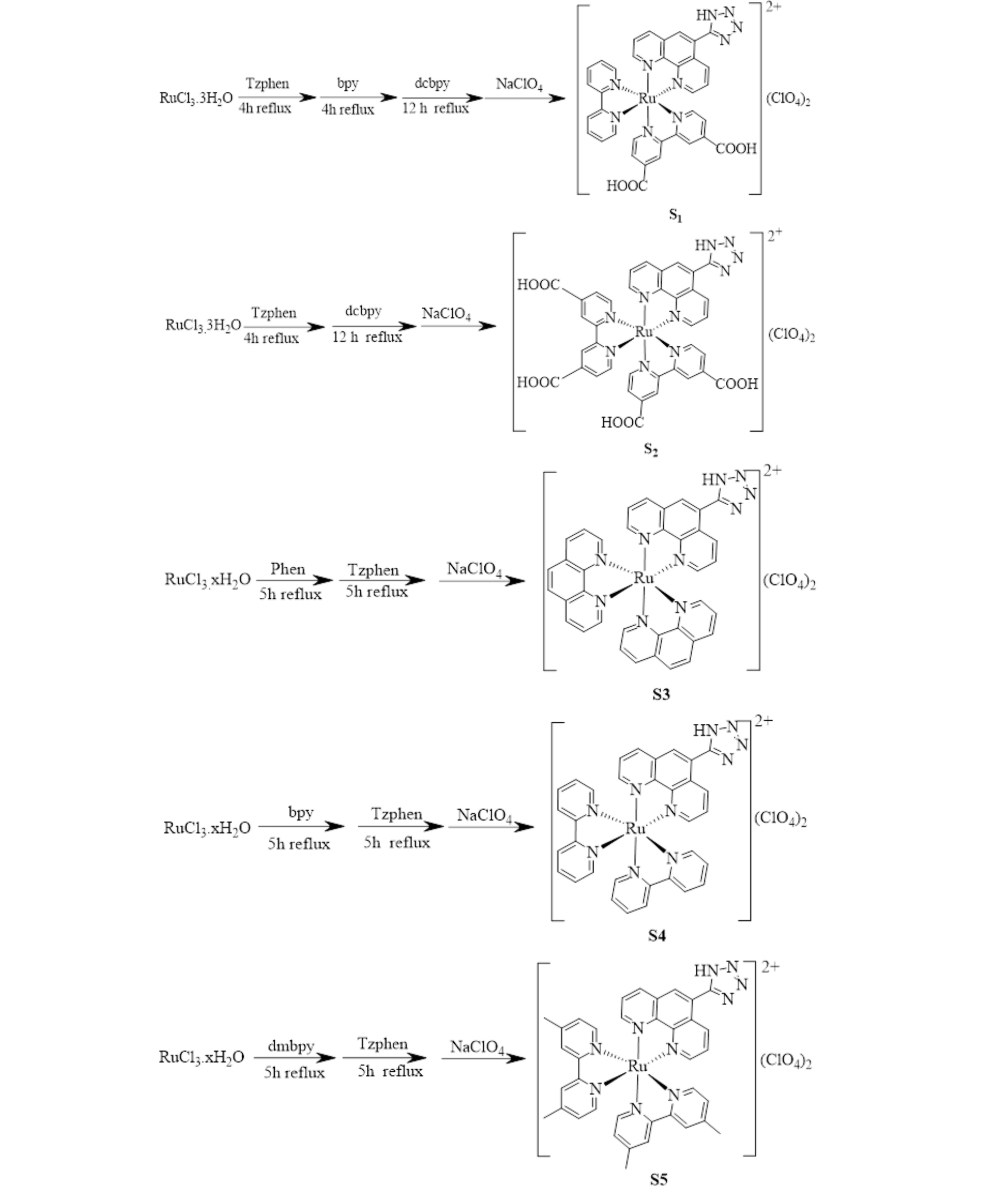
The synthesis route of complexes [Ru(Tzphen)(bpy)(dcbpy)]+2 (S1) - [Ru(dmbpy)2(Tzphen)]+2 (S5)

The complexes were synthesized according to literature-described methods (28, 29). The schematic of their synthesis route is shown in Figure 3. The ^1^HNMR and ^13^CNMR data and spectra are shown in SI.

#### 3.1.3. Characterization

IR spectra were recorded on a Perkin-Elmer 597 spectrometer. ^1^H NMR and ^13^C NMR spectra were recorded using a Bruker 400 MHz spectrometer with tetramethylsilane (TMS) as the internal standard. The cyclic voltammetry was measured with a SAMA500 electrochemistry system. Ultraviolet-visible (UV-Vis) absorption spectra were obtained using Ultrospec3100 pro spectrophotometer in CH_3_CN solution.

### 3.2. Cell Cytotoxicity

The B16F10 cells were placed in a 96-well plate and kept in a humid incubator with 5% CO_2_ and a temperature of 37°C. They were provided with a complete RPMI-1640 culture medium. Cells were incubated for 24 hours to adhere to and achieve applicable compliance. The cells that were attached were examined with varying concentrations of complexes. The previous culture medium was replaced with a culture medium containing MTT 24 to 72 h after incubation and was incubated for four hours. Eventually, as alluded to, the MTT assay was applied to investigate cell viability. The same technique was repeated and compared with and without solvent complexes. DMSO and Sorenson’s phosphate were added, and afterward, the absorbance of formazan at 570 nm was registered by a microplate reader.

### 3.3. Cell Apoptosis Investigation

To assess the apoptosis-inducing capabilities of disparate complexes, the annexin V-FITC/PI apoptosis detection kit (Exbio, Czech Republic) was utilized to investigate cell apoptosis ratios. Lastly, the quantitative cell apoptosis analyses were conducted by flow cytometry (FCM) (MACS Quant 10, Miltenyi Biotech GmbH). After implanting the cells in the plates of 6 wells and overnight incubation, the top environment of the plate wells was altered with 2 mL of fresh medium containing IC_50_ concentration of all remedy groups. The cells were then incubated for 48 h.

### 3.4. Reactive Oxygen Species Formation

To determine the facility of the complexes preplanned in the reactive oxygen species (ROS) creation, B16F10 cells were implanted in 6 well plates for 48 h in the presence of complexes and H_2_O_2_ as a positive control. The cells were treated with a fluorescent marker, dichlorodihydrofluorescein diacetate (DCFH-DA) (10 μM). After 2 h of incubation, they were washed twice with PBS and resuspended in 500 μL PBS. Then, the generated ROS inside cells was perused by a flow cytometric analysis (BD Facscalibur, Franklin Lakes, New Jersey, United States) analyzed by the FL2-H Purchaser Filter (FITC). Also, a high concentration (HC) reached from MTT IC_50_ was selected.

### 3.5. Cellular Uptake Study

The samples were labeled with RhB as a fluorescent agent to study their adsorption efficiency. To study cell uptake, the fluorescent agent of the complexes (0.05 w / w RhB to fat) was added, and the RhB depletion was separated by the above filtration method (Amicon® tube, 30 kDa M Melipour, Germany), and B16F10 cells in the plates were placed in six wells and incubated for 24 h. The cells were then treated with RhB-labeled samples with concentrations in the ranges of 10, 100, 200, 400, 800, 1600, 3200, 6400, and 12800 μg/mL. After incubation for four h, the residual weaving was replaced with 1 mL of 4% formaldehyde, fixed for four hours, and scanned by flow cytometer (BDBX50, Olympus, Japan).

### 3.6. Fluorescence Microscopic Studies

In addition, the adsorption of the final RhB-labeled formula was studied using fluorescence microscopic to obtain fluorescence images; B16F10 cells were implanted in a multi-chamber slide with a density of 105 2 2 cells. The slide was left to incubate for 48 hours to allow the cells to attach. They were fixed for four hours after washing with PBS and treatment with 4% formalin solution. Then, the cells were washed again with PBS and incubated with 1 μg.mL^-1^ 4,6-diamidino-2-phenylindole (DAPI) and 0.01 V/V Triton X-100 solution for 15 minutes. Finally, fluorescence pictures were taken using a microscope with a DAPI filter.

### 3.7. Quantitative Measurement of Autophagy

To measure autophagic potency, the cells were stained and analyzed using MDC under the manufacturer’s convention. After that, the cells were washed three times to eliminate MDC in PBS and treated after 48 hours of incubation. The cells were then washed twice with PBS and stained with 0.05 mmol/L MDC at 37°C for 10 minutes and were analyzed by fluorescence imaging microscope to assess the level of autophagy.

## 4. Results and Discussion

### 4.1. Synthesis and Characterization

The synthesis of ligands and corresponding complexes was conducted according to literature methods (28-34). The structures of the ligands and complexes were investigated and characterized by the ^1^H NMR, ^13^C NMR, UV-Vis absorption, cyclic voltammetry, and FT-IR. The results are tabulated in Table 1. 

**Table 1.** Photophysical and Electrochemical Properties of [Ru(Tzphen)(bpy)(dcbpy)]^+2^ (S1) - [Ru(dmbpy)_2_(Tzphen)]^+2^ (S5) Complexes

Complex	Absorbance λ (nm)	Emission λ_max_ (nm)	E_ox_ (Ru^II^/Ru^III^) (V)	E_red_^1/2^ (V)	E_gap_ (eV)	E_HOMO_ (eV)	E_LUMO_ (eV)
**S1**			+1.47	-0.995	2.46	-5.69	-3.23
**S2**			+1.29	-1.03, -1.48	2.32	-5.73	-3.41
**S3**	456	618	+1.36	-1.30, -1.45	2.29	-6.08	-3.79
**S4**	447	629	+1.34	-1.31, -1.52	2.26	-6.15	-3.89
**S5**	460	659	+1.28	-1.13, -1.51	2.19	-6.16	-3.97

### 4.2. Assessment of Cytotoxic Activity of Complexes In Vitro

To investigate the cytotoxicity of S1 - S5 complexes, cancer cells (B16F10, Hela, A549, BEL-7402, SIHA, SGC-7901) and LO2 cells of human normal liver cells were used. The IC_50_ values described in Table 2 indicate the lack of cytotoxic activity of the ligand against B16F10, A549 BEL-7402, SiHa, SGC-7901, and LO2 cells. Due to the greater hydrophobicity of the Tzphen sub-ligand than phen and dpp, the cytotoxic inactivity of S1 - S3 complexes against A549 and SiHa cell lines is evident. As a result, the S5 and S4 complexes enter the cells under the same conditions as the S1 - S3 complexes. The cytotoxicity of S5 and S4 complexes was significant compared to most selected cancer cells. They demonstrated little cytotoxic activity against normal hepatocyte LO2 cells. The cytotoxic activity of S1 - S5 complexes was comparable to that of B16F10 cells but less than that of cisplatin as a control under the same conditions. As a result, cytotoxicity activity increased with ligand attachment to metal and formation of metal complexes; and, due to the moderate cytotoxic effect of all S1 - S5 complexes on B16F10 cell growth, this cell was selected for testing.

**Table 2.** IC_50_ (μM) Values of the Complexes [Ru(Tzphen)(bpy)(dcbpy)]^+2^ (S1) - [Ru(dmbpy)_2_(Tzphen)]^+2^ (S5) on the B16F10, Hela, A549, BEL-7402, SIHA, SGC-7901 and LO2 Cell Lines

Complex	B16F10	HeLa	A549	BEL-7402	SiHa	SGC-7901	LO2
**Tzphen**	> 200	75 ± 1.9	> 200	> 200	> 200	> 200	> 200
**S1**	25.5 ± 1.7	> 200	> 200	> 200	> 200	> 200	> 200
**S2**	45.3 ± 2.1	36.8 ± 1.3	> 200	> 200	> 200	68 ± 3.2	> 200
**S3**	38.1 ± 2.5	> 200	> 200	50 ± 1.6	> 200	> 200	> 200
**S4**	32.6 ± 1.9	45.5 ± 1.5	48.3 ± 1.9	28 ± 2.6	58 ± 3.2	> 200	> 200
**S5**	28.5 ± 3.1	35.2 ± 2.9	35.2 ± 1.9	18 ± 2.8	> 200	> 200	> 200
***cis*** **-platin**	25.2 ± 2.3	8.5 ± 0.7	6.2 ± 1.1	13.1 ± 0.9	n.d	n.d	n.d

### 4.3. Reactive Oxygen Species Generation

**Figure 4.**
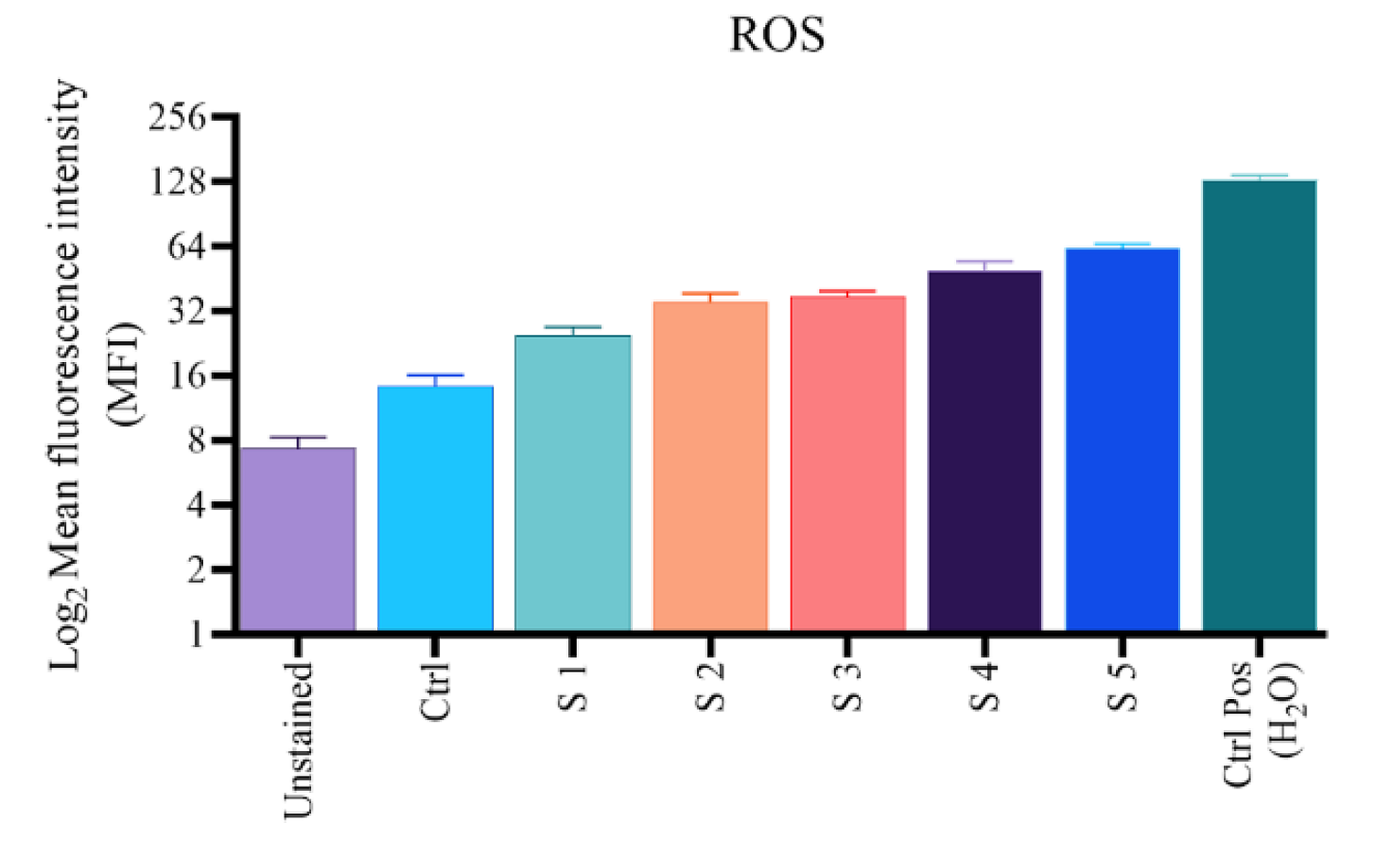
Intracellular reactive oxygen species levels in B16F10 cells

To confirm the ROS production within the cells, the DCFH-DA fluorescent probe was exerted. Figure 4 shows a five-fold increase in DCF fluorescent intensity compared to control for S1 - S5 complexes after treatment of B16F10 cells with 25 mM for 24 hours. Due to the higher production and lesser utilization of intracellular ROS levels of the S1 complex and, to some extent, S2, the S5 complex likely causes the highest increase in ROS levels because the intracellular ROS level depends on the balance of ROS production and utilization of the complexes. The overall result suggests that the increase in intracellular ROS levels is due to the presence of complexes.

### 4.4. Apoptosis Method with Annexin V/PI Staining Method

**Figure 5.**
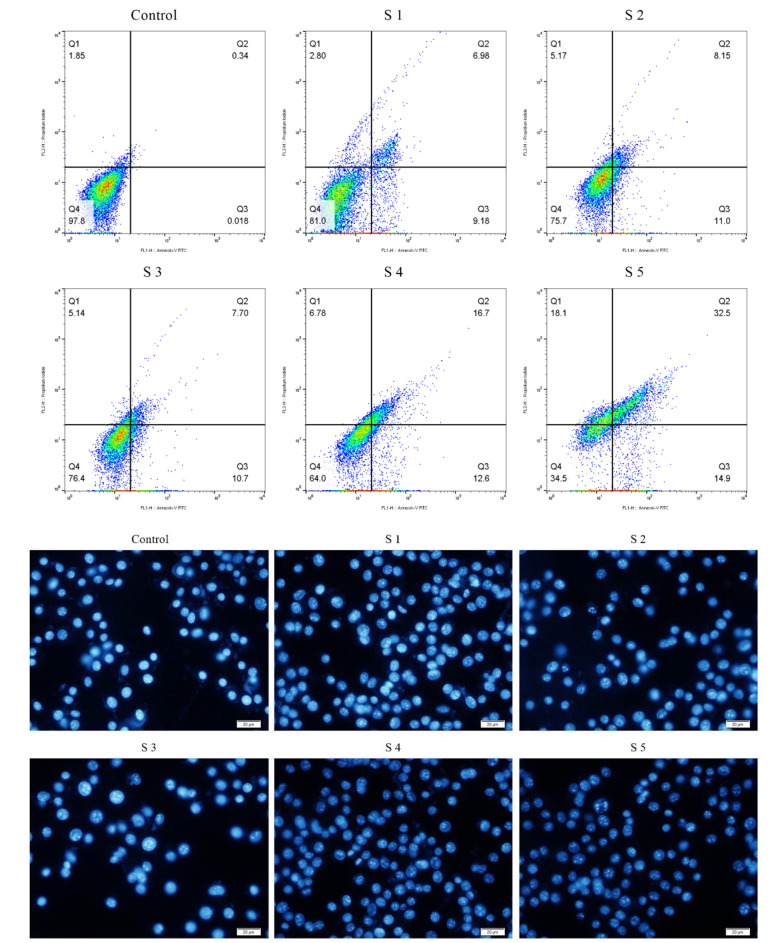
Top, apoptosis in B16F10 cells; bottom, the percentage of apoptotic cells

Flow cytometry was assessed to evaluate cell apoptosis induced by S1 - S5 complexes using the annexin V/PI staining method and morphological evolution of cells with orange acridine (AO)/ethidium bromide (EB) impregnated cells. Quantitative results showed that the complexes could cause apoptosis in B16F10 cells. In particular, compared to cells treated with S1 - S5 complexes, cells treated with S5 had the highest apoptotic percentage (Q3) of 14.9% compared to control. This percentage was 9.18%, 11%, 10.7%, and 12.6% for S1 - S4, respectively. In brief, the cell apoptosis method showed that the effect of treating B16F10 cells with 25 mM complexes of S1 - S5 for 24 hours could be mainly due to apoptotic features. For instance, cell composition, nucleation, and chromatin density are attributed. To evaluate and confirm the claim that S4 and S5 complexes can increase cell uptake or transfer, the uptake of these complexes was examined by flow cytometric analysis (Figure 5). 

### 4.5. Cell Uptake Studies

**Figure 6.**
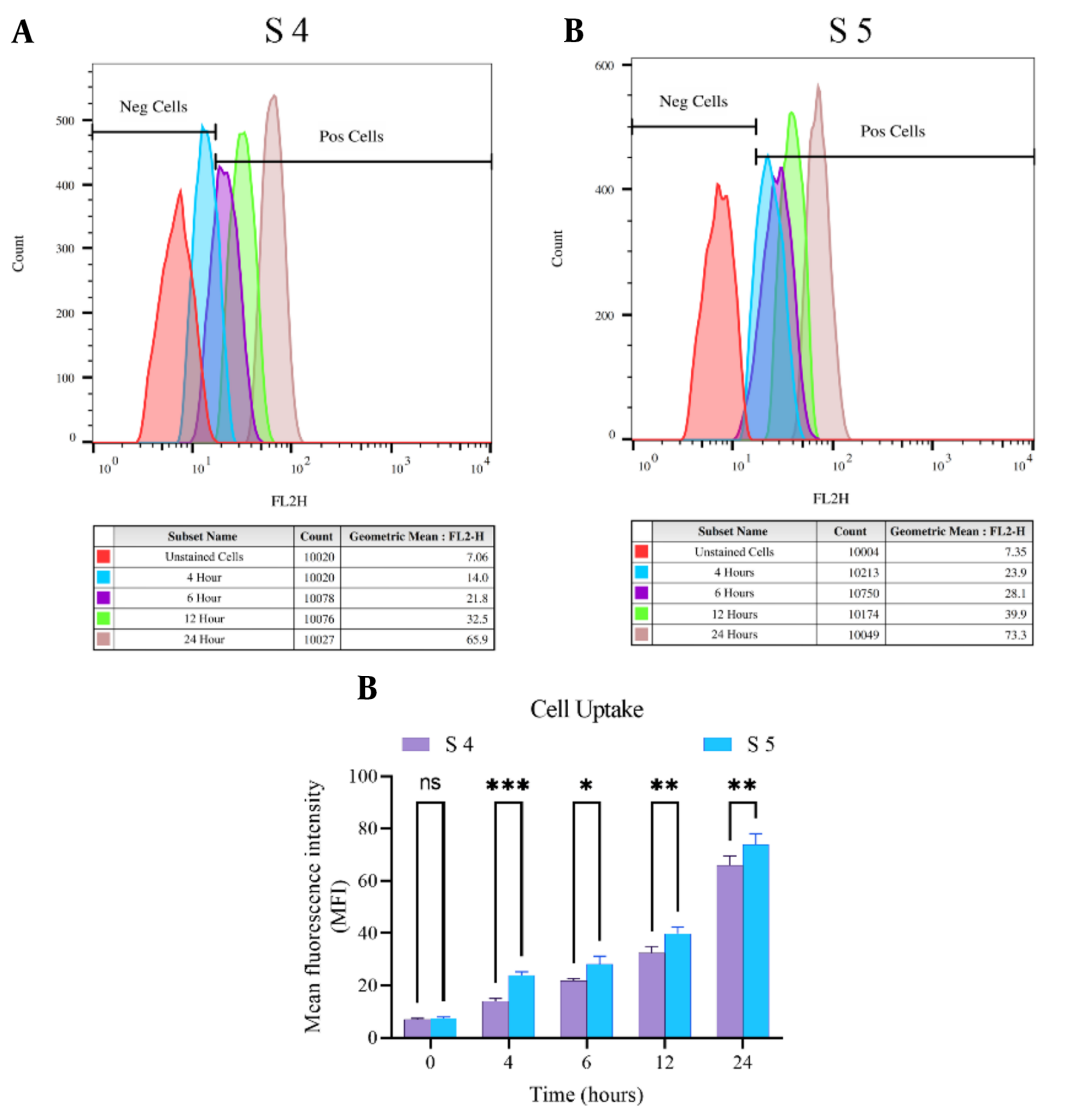
Cellular uptake survey: Schema chart of A, [Ru(Tzphen)(bpy)_2_]^+2^ (S4); B, [Ru(dmbpy)_2_(Tzphen)]^+2^ (S5) evaluated by FCM; and C, statistics curve of uptake rates of S4 and S5 analyzed by flow cytometry

To evaluate and confirm that S4 and S5 complexes can increase cell uptake or transfer, the uptake of these complexes was examined by flow cytometric analysis. After four hours of incubation, the cells were washed and imaged with a fluorescence imaging microscope to detect the intensity of cell uptake. The cell adsorption of the complexes showed that the S4 and S5 complexes had higher cell adsorption, and S5 had better internalization and higher fluorescence light intensity than S4 (Figure 6). 

### 4.6. Autophagy in the B16F10 Cells

**Figure 7.**
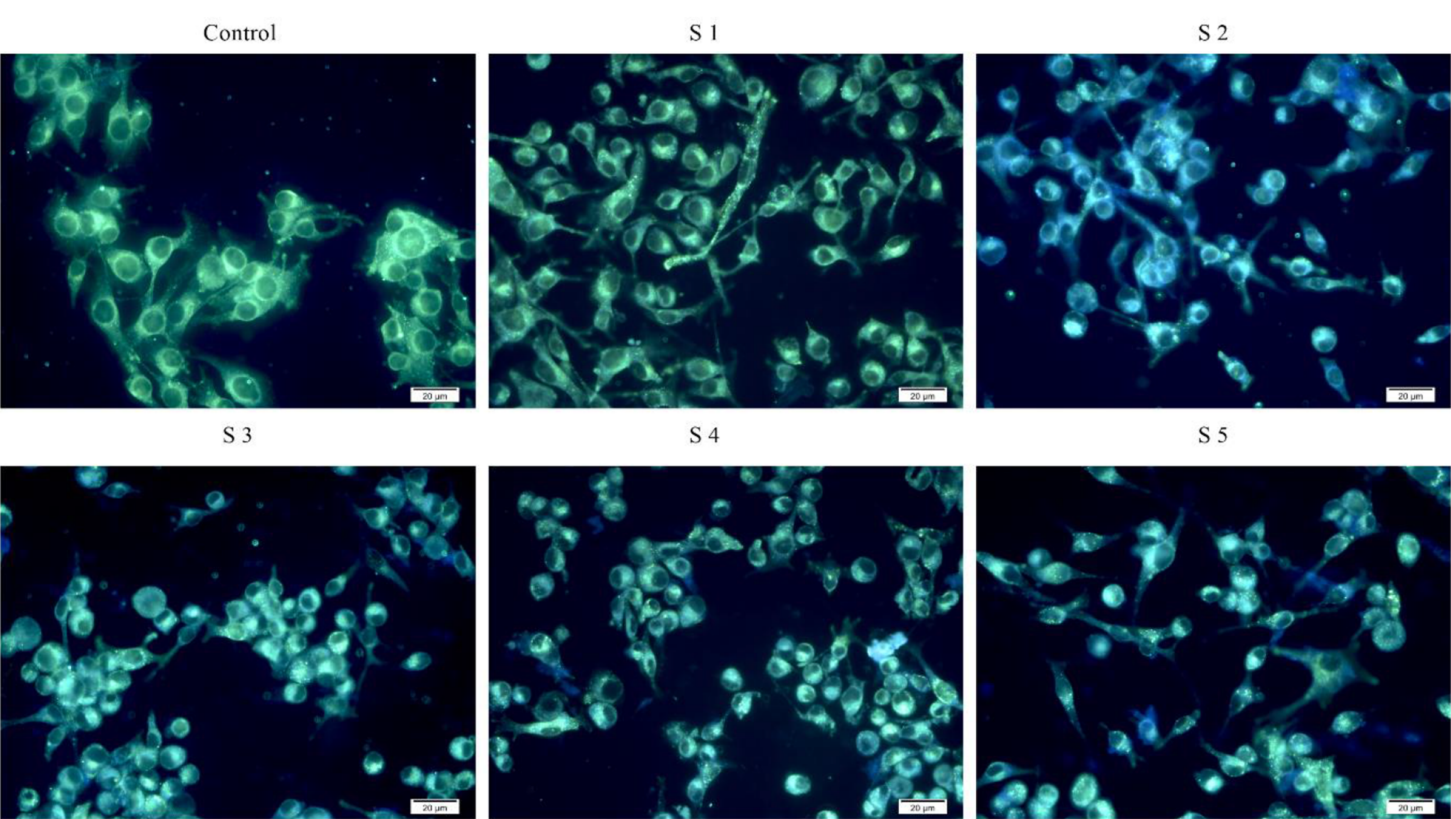
Elevated autophagy in B16F10 cells treated with the [Ru(Tzphen)(bpy)(dcbpy)]^+2^ (S1) - [Ru(Phen)_2_(Tzphen)]^+2^ (S3) complexes

The autophagy surface of the B16F10 cells treated with the S1-S3 complexes was stained using Cadaverine Monodensil (MDC), a green fluorescent probe, which labels the vacuolar components of the autophagy pathway. As shown in Figure 7, the intensity of MDC fluorescent in B16F10 cells treated with the S1 complex (170.81) was stronger than that treated with S2 (2.72) and S3 (3.83) complexes compared to the control. The intensity of MDC fluorescence increased significantly as the number of autophagy cells increased, indicating that the intensity of MDC fluorescence was dependent on the effects of autophagy and that this effect was a concentration-dependent approach.

## 5. Conclusion

In this project, we developed five Ru(II) complexes based on 1,10-phenanthroline ligand containing tetrazole substitution, S1, S2, S3, S4, and S5 promoted cancer treatment. This type of complex can enhance the generation of free radicals, cytotoxic activity, and also cell apoptosis. MDC staining display complexes could induce autophagy, and the intensity of MDC fluorescent in B16F10 cells treated with the S1 complex (170.81) was stronger. Moreover, the cell adsorption of the complexes demonstrated that the S4 and S5 complexes had higher cell adsorption, and S5 had better internalization and higher fluorescence light intensity than S4.
