# Erratum for Ruthenium (II) Complexes Based on Phenanthroline-Tetrazole as Possible Anticancer Agents [Iran J Pharm Res. 2023;22(1): e136738]

**DOI:** 10.5812/ijpr-157768

**Published:** 2024-11-26

**Authors:** Saeid Abaspour, Behzad Soltani, Hamed Hamishehkar, Moayad Hossaini Sadr

**Affiliations:** 1 Chemistry Department, Faculty Science, Azarbaijan Shahid Madani University, Tabriz, Iran; 2 Drug Applied Research Center, Tabriz University of Medical Sciences, Tabriz, Iran

We would like to correct an error in the published article regarding the affiliation of the corresponding author (1). This mistake originated from the authors during the manuscript submission process. The correct affiliation of the corresponding author is as follows:

Chemistry Department, Faculty of Science, Azarbaijan Shahid Madani University, Tabriz, Iran.

We sincerely apologize for this oversight and any confusion it may have caused. We take full responsibility for the error and appreciate your understanding.

Sincerely Yours,

Dr. Elham Rezaee

School of Pharmacy, Shahid Beheshti University of Medical Sciences, Tehran, Iran

Editor-in-Chief of IJ Pharmaceutical Research
